# Prediction of ultra-high-order antibiotic combinations based on pairwise interactions

**DOI:** 10.1371/journal.pcbi.1006774

**Published:** 2019-01-30

**Authors:** Itay Katzir, Murat Cokol, Bree B. Aldridge, Uri Alon

**Affiliations:** 1 Department of Molecular Cell Biology, Weizmann Institute of Science, Rehovot, Israel; 2 Axcella Health, Cambridge, MA; 3 Laboratory of Systems Pharmacology, Harvard Medical School, Boston MA; 4 Department of Molecular Biology and Microbiology, Tufts University School of Medicine, Boston, MA; 5 Department of Biomedical Engineering, Tufts University School of Engineering, Medford, MA; ETH Zurich, SWITZERLAND

## Abstract

Drug combinations are a promising approach to achieve high efficacy at low doses and to overcome resistance. Drug combinations are especially useful when drugs cannot achieve effectiveness at tolerable doses, as occurs in cancer and tuberculosis (TB). However, discovery of effective drug combinations faces the challenge of combinatorial explosion, in which the number of possible combinations increases exponentially with the number of drugs and doses. A recent advance, called the dose model, uses a mathematical formula to overcome combinatorial explosion by reducing the problem to a feasible quadratic one: using data on drug pairs at a few doses, the dose model accurately predicts the effect of combinations of three and four drugs at all doses. The dose model has not yet been tested on higher-order combinations beyond four drugs. To address this, we measured the effect of combinations of up to ten antibiotics on *E*. *coli* growth, and of up to five tuberculosis (TB) drugs on the growth of *M*. *tuberculosis*. We find that the dose model accurately predicts the effect of these higher-order combinations, including cases of strong synergy and antagonism. This study supports the view that the interactions between drug pairs carries key information that largely determines higher-order interactions. Therefore, systematic study of pairwise drug interactions is a compelling strategy to prioritize drug regimens in high-dimensional spaces.

## Author summary

Drug combinations are important to increase efficacy and reduce resistance of treatment for infection and cancer. The major challenge is the vast number of experiments needed to scan the space of combination in order to find rare synergistic drugs and their optimal doses. In the past few years there has been an advance in the ability to predict the effects of drug cocktails, using a small number of experiments on drug pairs. These approaches have not been tested on combinations of more than a few drugs. Thus, it remains unclear whether there are useful combinations of 5–10 drugs that work at low doses. Here we show that a mathematical model can use data for drug pairs to predict ultra-high-order cocktails for *E. coli* and an important pathogen, *M. tuberculosis*. (1) We measured the effect of 124 new combinations of 3–10 antibiotic drugs, each at 13 doses. (2) We show that our model accurately predicts the effect of these cocktails using pair measurements alone. (3) We predict and verify new high-order combinations for TB that provide high efficacy at low doses, overcoming the well-known problem in TB that each drug alone has low efficacy.

## Introduction

Drugs in combination, compared to single drugs, are attractive therapies because of their potential for increased potency and slowed acquisition of drug resistance [1–14]. Combination therapy is important in cases where single drugs are not effective enough at tolerable doses, such as in the case of cancer and TB. In TB, the standard of care includes combinations of three or four drugs. For example, one current regimen for drug-sensitive TB uses a four-drug combination for two months, followed by a two-drug combination for at least four months [15]. Drug-resistant TB therapy is more arduous, involving long-term, high-order combinations of antibiotics with more severe side effects [16].

It is therefore of interest to find additional combinations of drugs which are effective at low doses. However, it is difficult to experimentally test all possible combinations. This is because the number of experiments needed grows exponentially with the number of drugs and doses, a challenge known as the combinatorial explosion problem [17–20]. For example, if there are N = 10 drugs at D = 10 possible doses, there are D^N^ ~10^10^ combinations. Hence, effective low-dose cocktails of multiple drugs might exist but cannot be found by exhaustive experimental search.

To overcome the combinatorial explosion problem, one approach is to measure a small number of combinations and use mathematical models to predict the rest. Recent studies explored the possibility of measuring only drug pairs to predict triplets and higher-order cocktails. Using pairs involves much fewer experiments than an exhaustive screen: the number of experiments scales quadratically with the number of drugs/doses rather than exponentially. For example, Wood et al [21] provided a formula based on single and pair measurements that gave excellent predictions for up to 4 antibiotics in *E. coli*. This formula was less accurate in datasets with larger experimental noise [22]. An alternative approach, called the pairs model, is more noise-resistant but less precise [23]. These approaches can predict high-order cocktails only at the same doses at which the pairs and single drugs are measured. They therefore preclude a computational scan over all possible doses, which is important for finding effective cocktails.

A recent advance, the dose model [22], provides excellent predictions for all doses based on measuring singles and pairs at a small number of doses, even in the presence of sizable experimental noise. For example, for ten drugs, the dose model typically requires measuring the 45 possible pairs at about 10 dose combinations. This reduces the number of measurements needed from 10^10^ to about 500.

The dose model works by assuming that each drug i changes the effective concentration of the other drug j in a way that is described by an interaction parameter, a_ij_. The model uses measurements of pairs at a few dose combinations to estimate the parameters a_ij_ and a_ji_ for each pair. It then uses these parameters to predict high-order combinations of three or more drugs (Methods).

The dose model was tested so far only on triplets and quadruplets of antibiotics and a triplets of cancer drugs [22]. It is therefore unclear whether the dose model can predict interactions for higher-order combinations, which can potentially include more high-order interactions that are not captured at the level of pairs. Here we ask whether the dose model can predict combinations of up to ten drugs, using antibiotic effect on *E*. *coli* and the clinically relevant pathogen *M*. *tuberculosis*. We find that in both *E*. *coli* and *M*. *tuberculosis*, the dose model provides excellent predictions for high-order combinations. The dose model identifies several synergistic combinations of TB drugs.

## Results

### The dose model predicts well the effect of combinations of 3–10 antibiotics on *E*. *coli* growth

To test the dose model, we chose 10 antibiotics with diverse mechanisms of action (Table 1). We define the effect of the drugs, g, as the reduction in growth of *E*. *coli* relative to growth with no drugs, evaluated by the optical density (OD 600nm) after 12h of growth in the presence of the drugs.

**Table 1:** The antibiotics used in this study, their abbreviations, target processes, and the top dose.

#	drug	abbreviation	target process	Top dose (ug/ml)
1	ampicillin	AMP	betalactam penicillin	8
2	azithromycin	AZI	50S	5
3	aztroenam	AZT	monobactam	0.04
4	chloramphenicol	CHL	protein synthesis chain elongation	2.6
5	irgasan	IRG	fatty acid synthesis	0.9
6	meropenem	MER	betalactam	0.2
7	moxifloxacin	MOX	DNA gyrase	0.14
8	rifampicin	RIF	RNA polymerase	7
9	spectinomycin	SPE	30S	18
10	tetracycline	TET	30S	1

**Fig 1:**
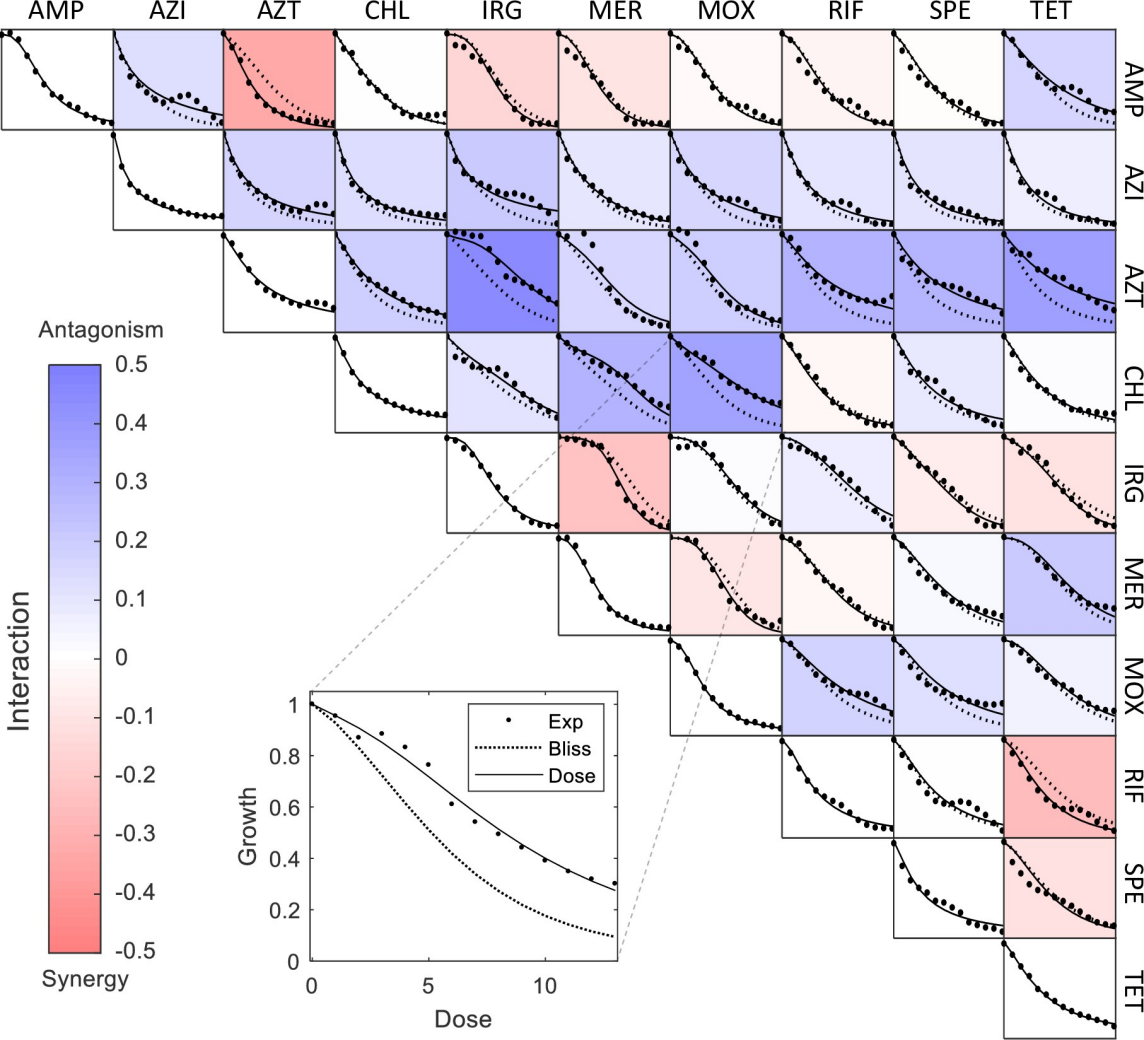
Effect of single and pairs of ten antibiotics on E coli growth was measured to determine the dose model parameters. The panels on the diagonal shows the relative growth reduction of *E*. *coli* grown with individual antibiotics (Table 1) at 13 doses (dots). Doses for each drug were spaced linearly such that the halfway effect D50 is approximately at the middle dose. These dose-response curves are well described by Hill curves (solid line). The off-diagonal panels show the two-drug responses for all 45 pairs of the ten drugs, at 13 dose combinations (dots). For example, the panel located at the 4^th^ row and 7^th^ column shows the pair response of drug 4(CHL) and drug 7(MOX) (also shown in the inset figure). We mixed the drugs at the 13 doses used for the single-drug dose-response curves, thus diluting each drug by a factor of two. The pair response curves were used to determine the dose model parameters (a_ij_). After fitting the parameters, the response curves of the pairs are well described by the dose model (solid lines). The pair interactions strength I = log(g12-g1g2+1), which indicates the mean deviation from the Bliss model (dashed line) is visualized by the color of each panel. The response curves presented here are the average of two (for combination 1–28) or three (for combination 29–115) biological replicates.

We first evaluated the dose-response curves of individual antibiotics at 13 doses (Fig 1, panels on the diagonal). Doses for each drug were spaced linearly such that the halfway effect D50 is approximately at the middle dose. These dose-response curves were well described by Hill curves (Methods) [24], [25] (R^2^>0.95) with Hill coefficients in the range n = 0.95–3.25.

We next measured two-drug responses for all 45 pairs of the ten drugs, at 13 dose combinations. Here we used the method of Cokol et al [26], by using “diagonal” dose combinations: we mixed the drugs at the 13 doses used for the single-drug dose-response curves, thus diluting each drug by a factor of two. Thus, for each of the 45 drug pairs, we sampled the two-drug response surface at 13 dose combinations on the diagonal.

Fig 1 shows the 45 drug pair measurements, and compares them to the Bliss independence model [27], [28] in which the effects (relative reduction in growth) of the two individual drugs is multiplied (Methods). Bliss independence was shown to be a reasonable model for high-order drug combinations, more than other baseline models such as the Loewe formula[29].

Deviations from Bliss indicate drug interaction, namely synergy and antagonism. Similarly to [30]-[31], we calculated the interaction strength for all pairs by the logarithm of the deviation from the Bliss model, I = log2(g12-g1*g2+1) (visualized by color in Fig 1). Note that this formula is asymmetric, with higher |I| for synergy than antagonism, especially when interactions are strong. This asymmetry is small (<5%) for most (95%) of the interactions in this study.

We find that 17 of the drug pairs were antagonistic, 7 were synergistic and 21 showed no appreciable interaction within experimental error (zero interaction is within the 95% confidence interval, see S1 Fig).

We used these measurements to calibrate the dose model formula (Methods), by estimating interaction parameters for each pair, a_12_ and a_21_, which describe how drug 1 affects the dose of drug 2 and vise-versa:

$$D_{1eff}=\frac{D_{1}}{\left(1+a_{12}\frac{D_{2eff}/D_{02}}{1+D_{2eff}/D_{02}}\right)};D_{2eff}=\frac{D_{2}}{\left(1+a_{21}\frac{D_{1eff}/D_{01}}{1+D_{1eff}/D_{01}}\right)}$$

The responses of the pairs are well described by the dose model (Fig 1, R^2^ = 0.9–0.98).

With the interaction parameters a_ij_ for each pair, we next used the dose model to predict the effect of all combinations of the ten drugs (Methods).

To test the predictions, we experimentally tested higher-order combinations. We chose 115 combinations of three to ten drugs (S1 Table) that span the range of predicted synergy and antagonism. The combinations tested included 27 triplets, 25 quadruplets, 16 quintuplets, 12 combinations of 6, 7 and 8 drugs, all ten 9-drug combinations and the full combination of the ten drugs.

**Fig 2:**
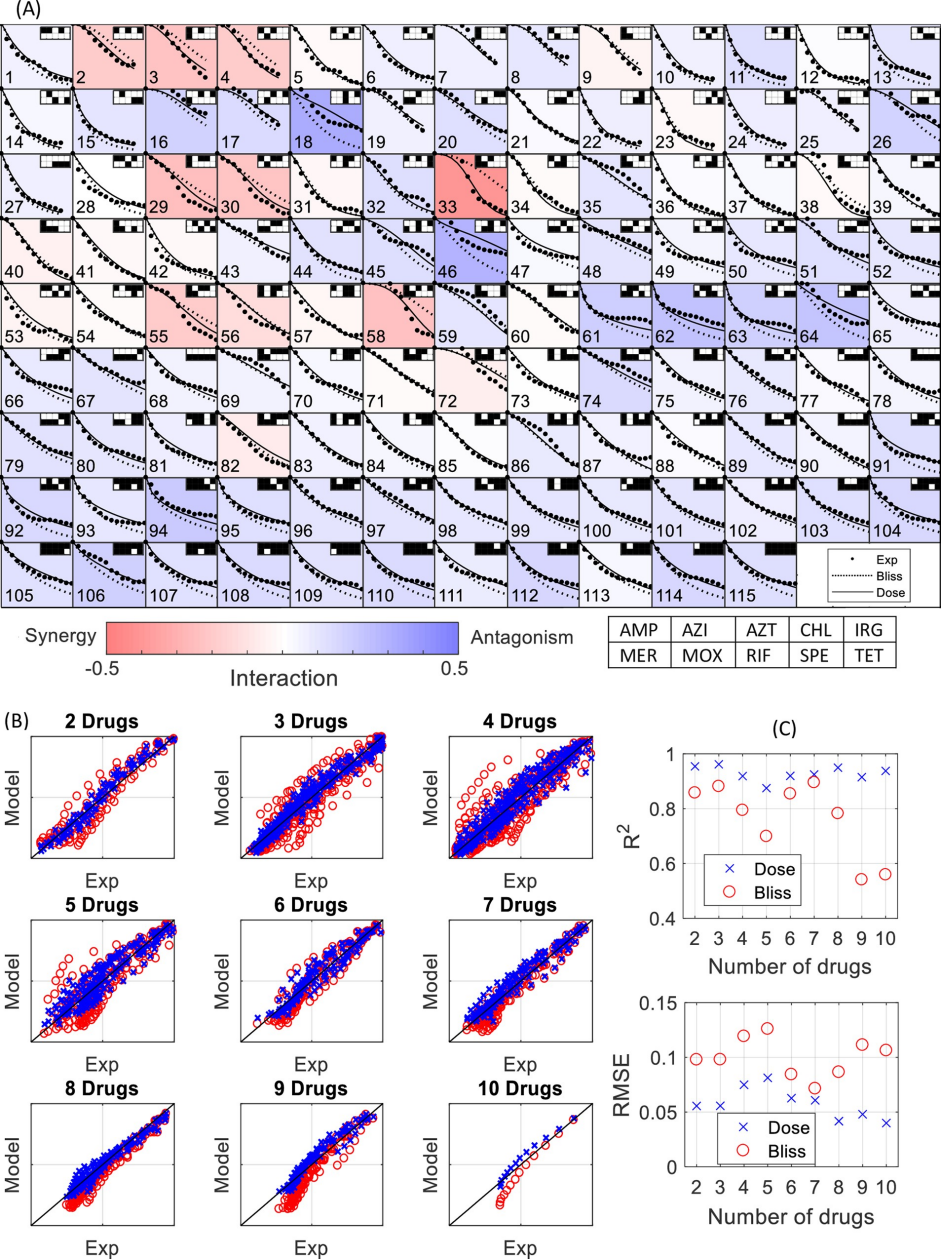
The dose model predicts well the effect of combinations of 2–10 antibiotics on *E*. *coli* growth. (A) The response curves for all 115 combinations (dots) compared to the dose model predictions (solid lines) and the bliss model predictions (dashed lines). The Interactions strength I = log(g12-g1g2+1), which indicates the mean deviation from the Bliss model (dashed line) is visualized by the color of each panel. The black and white squares on the top-right corner of each panel indicated which drugs where included in the cocktail. For example, in the first panel drugs AMP(1), MER(6) and MOX(7) were included in the cocktail. (B) The normalized growth compared to the dose model (x) and the Bliss model (o) predictions for 2–10 antibiotics. (B) R^2^ and RMSE values for the Dose model (x) and the Bliss model (o). The combination legend is also in S1 Table. The response curves presented here are the average of two (for combination 1–28) or three (for combination 29–115) biological replicates.

For each combination, we measured the effect at 13 diagonal dose combinations (dots in Fig 2A). Thus, the dose combinations mixed the 13 doses used for the response curve, diluting each drug by a factor M for an M-drug combination.

We find that the high-order combinations show sizable interactions when compared to the Bliss model, with R^2^ agreement in the range 0.5–0.85 (Fig 2B and 2C). At nine and ten drugs Bliss predictions were generally poor, with R^2^ = 0.6, indicating sizable interaction. We find, in agreement with Russ et al[29], that the Bliss multiplicative null model is better than the Loewe [32] null model for high-order combinations (S2 Fig and S3 Fig).

In contrast to the null models, the dose model shows excellent agreement for any number of drugs, with R^2^ in the range 0.88–0.96 and RMSE of 0.04–0.07. Remarkably, it makes excellent prediction for nine and ten drugs (R^2^>0.9).

We also compared the model residual distribution (the difference between model and measurements) to the experimental noise distribution (estimated from the difference between measurements in different biological repeats). We find that the dose model residual distribution is very similar to the experimental noise distribution, with the RMSE of the model residuals being slightly smaller (0.077 for the model and 0.078 for the experimental noise). In contrast the residuals of the Bliss or Lowe models are very different from the noise distribution (RMSE of 0.113 and 0.173), indicating model errors beyond experimental noise (S4 Fig).

We next asked whether there are specific combinations that significantly deviate from the dose model predictions. Such combinations can offer an example of high-order interactions that cannot be accounted for using the pair interactions in the dose model. We calculated the RMSE values of the model for each of the 115 combinations and compared it to the RMSE distribution evaluated from experimental noise (S5 Fig). We found that there is no significant difference between the two distributions in contrast to the RMSE distribution of the Bliss or Loewe models.

Additionally, we performed t-test for the mean difference between the model and the experiment for each of the 115 combinations (two or three repeats for each). None of the combinations showed a significant difference after correcting for multiple hypothesis testing errors by means of FDR [33] with 95% confidence. Although there is no combination for which the model deviate beyond the statistically expected deviation, one can test by repeated experiments the most deviating combinations to increase the statistical power and search for statistically significant combinations. In order to identify combinations for further study we made a list of the RMSE values for each of the combinations, ordered by the difference between the model error and the experimental error (see S2 Table). One can also find the residual plots (S6 Fig) useful to identify potential combinations.

After testing the model’s predictive power, we sought to search for synergistic drug combinations. We used the model to numerically navigate the space of drug-dose combinations. We asked what fraction of combinations of 3–10 drugs from the current drug panel are predicted to show synergy or antagonism. We find that predicted synergy is rare in cocktails above four drugs, with antagonism being much more common (S7A Fig). This stems from the fact that most of the pairs in the present study are antagonistic. This result is general for the dose model and does not depend on the choice of the experimental design (see S7B Fig). Simulations of a hypothetical scenario in which we set most drug pairs to be synergistic result in mostly synergistic high-order combinations (S8 Fig).

The most synergistic cocktail relative to the Bliss model in the present drug panel is predicted to be the combination Ampicillin(1), Aztroenam(3), Meropenem(6) and Moxifloxacin(7). This combination was among those that we tested experimentally (Fig 2, combination 33). It was indeed the most synergistic cocktail among the 115 combinations tested. It provided reduction of 50% in overall dose needed to achieve a 50% reduction in growth, g = 0.5 relative to the Bliss model.it is possible that the synergy relative to Bliss is a result of the fact that three of the four drugs (AMP, AZT, MER) are beta-lactams, which probably bind the same target. Hence, a more appropriate null model for comparison in this case is Loewe (as suggested in ref [34]). The interaction of this combination using the Loewe model as a null model is additive.

### The dose model predicts well high-order interactions in *M*. *tuberculosis*

We also tested the dose model for the effects of TB drugs on *M*. *tuberculosis*. Drug effect was defined by growth inhibition after 5 days of growth in the presence of the drugs normalized to growth with no drugs. We used nine TB drugs with various mechanisms of action (S2 Table)

**Fig 3:**
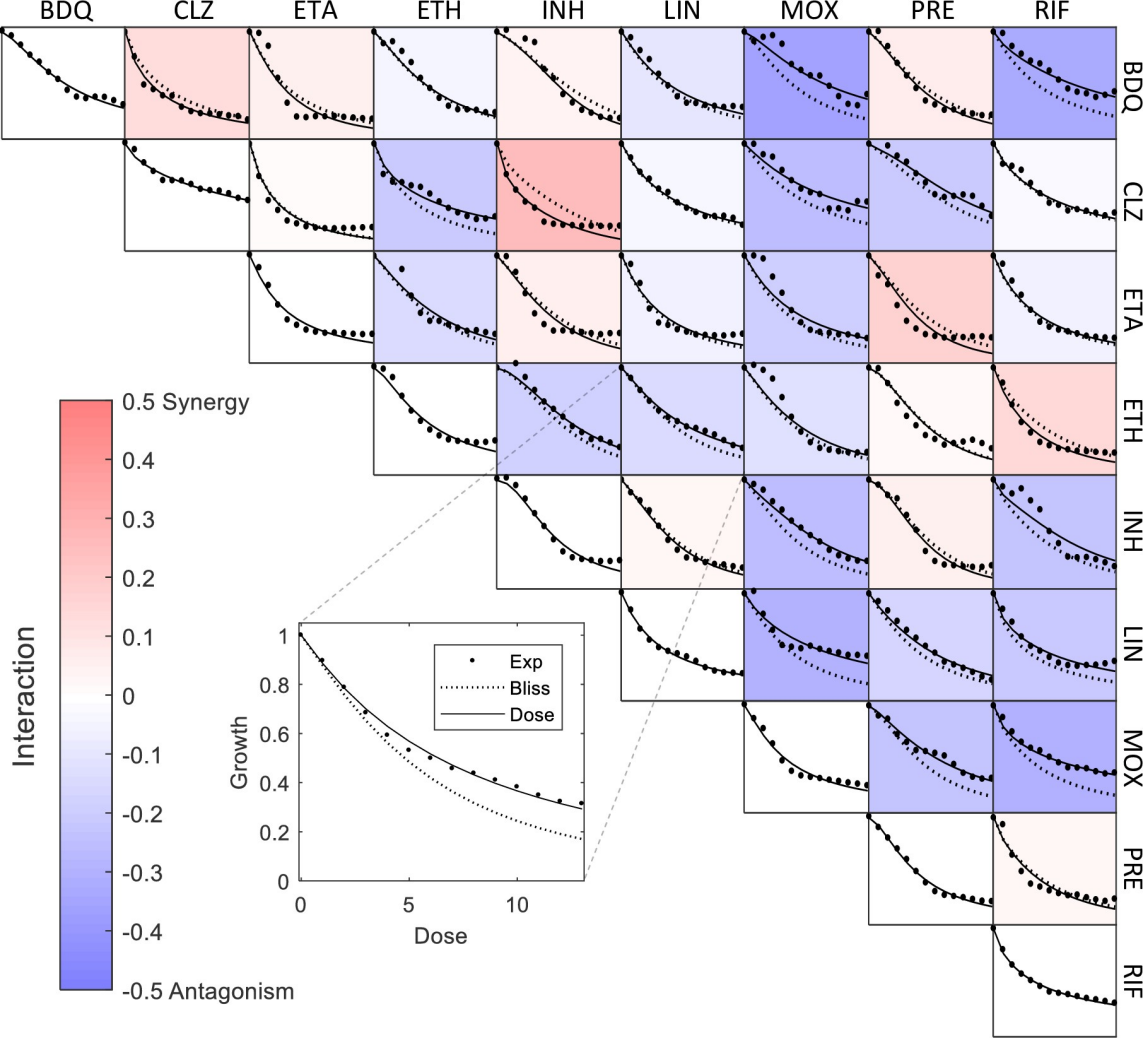
The effect of single and pair of 9 TB drugs on *M*. *tuberculosis* growth was measured to determine the dose model parameters. The panels on the diagonal shows the individual antibiotics (Table 2) response at 13 doses (dots). Doses for each drug were spaced linearly such that the halfway effect D50 is approximately at the middle dose. These dose-response curves were well described by Hill curves (solid line). The off-diagonal panels show the two-drug responses for all 36 pairs of the 9 drugs, at 13 dose combinations (dots). For example, the panel located at the 4^th^ row and 6^th^ column shows the pair response of drug 4 and drug 6 (also shown in the inset figure). We mixed the drugs at the 13 doses used for the single-drug dose-response curves, thus diluting each drug by a factor of two. The pair response curves were used to determine the dose model parameters (a_ij_). After fitting the parameters, the response curves of the pairs are well described by the dose model (solid line). The pairs interactions strength I = log(g12-g1g2+1), which indicates the mean deviation from the Bliss model (dashed line) is visualized by the color of each panel. The response curves presented here are the average of two biological replicates.

The single-drug dose-response curves were well described by Hill curves [24], [25] (R^2^>0.95) with Hill coefficients in the range n = 0.95–2.3. The responses of the drug pairs (experimental data originally published by Cokol et al [26]) were well described by the dose model (Fig 3, R^2^ = 0.85–0.98).

**Table 2:** The TB drugs used in this study, their abbreviations, target processes, and the top dose.

#	drug	abbreviation	Target process	Top dose (ug/ml)
1	bedaquiline	BDQ	ATP synthase	0.6
2	clofazimine	CLZ	DNA replication	2.8
3	ethionamide	ETA	Mycolic acid synthesis	3
4	ethambutol	ETH	Arabinogalactan synthesis	1.5
5	isonizaid	INH	Mycolic acid synthesis	0.18
6	linezolid	LIN	Protein synthesis	3
7	moxifloxacin	MOX	DNA gyrase	0.35
8	pretomanid	PRE	Protein synthesis	0.8
9	rifampicin	RIF	RNA polymerase	0.06

As above, we calculated the interaction strength I = log2(g12-g1g2+1) for all 36 pairs (visualized by color in Fig 3). We find that 16 of the pairs were antagonistic, and 6 were synergistic and 14 showed no appreciable interaction within experimental error (zero interaction is within the 95% confidence interval).

We used the interaction parameters a_ij_ to predict the effects of combinations of all combinations of drugs at all doses. Based on these predictions, we chose combinations predicted to be most synergistic and most antagonistic. We experimentally tested 9 TB drug combinations, including 4 triplets, 4 quadruplets and 1 quintuplet. Each combination was tested in the 13 diagonal dose combination, with two biological replicates.

**Fig 4:**
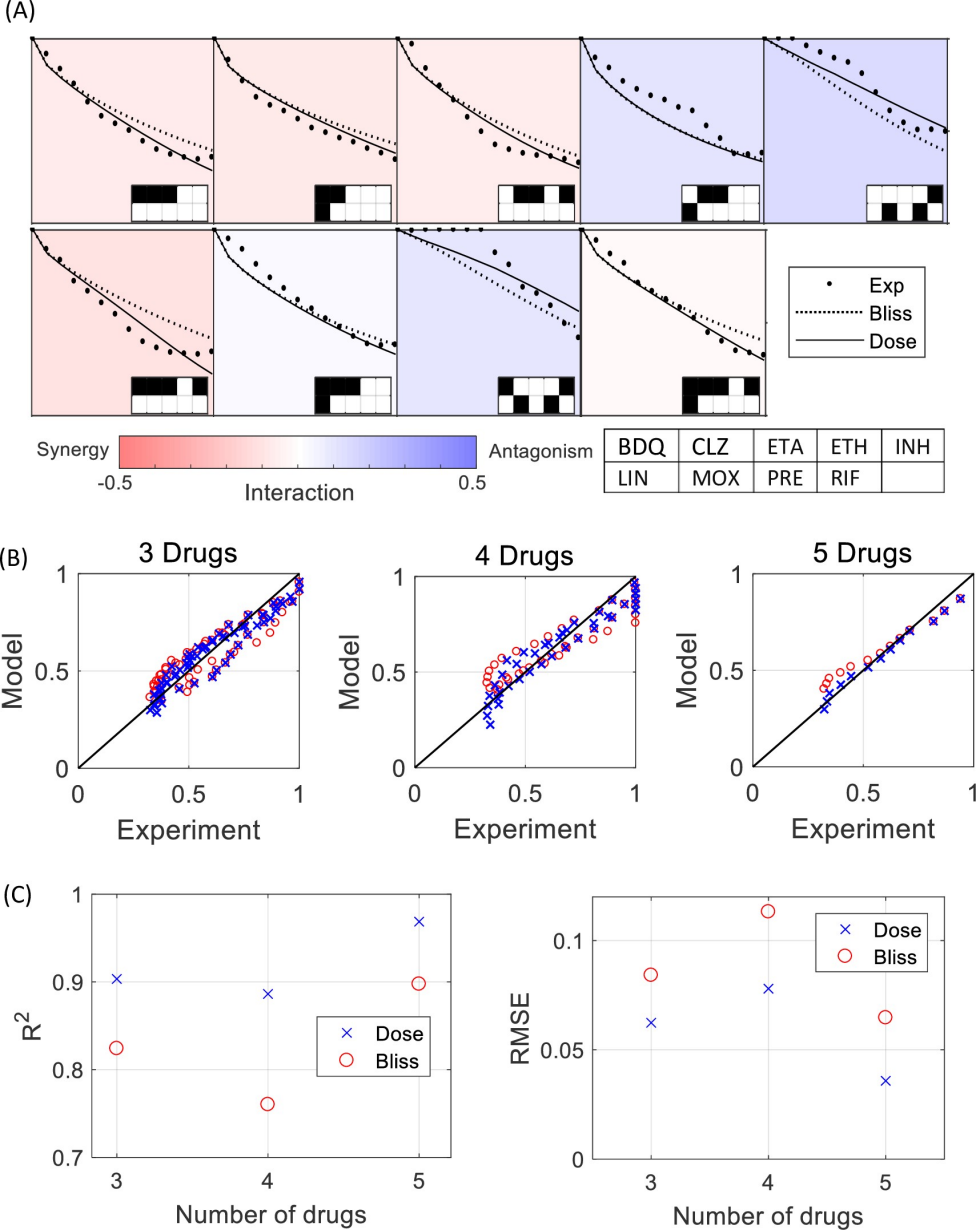
The dose model predicts well the effect of combinations of 3–5 TB drugs on *M*. *tuberculosis* growth. (A) The response curves for all 9 combinations (dots) compared to the dose model predictions (solid lines) and the bliss model predictions (dashed lines). The Interactions strength I = log(g12-g1g2+1), which indicates the mean deviation from the Bliss model (dashed line) is visualized by the color of each panel. (B) The normalized growth compared to the Dose model (x) and the Bliss model (o) predictions for 3–5 TB drugs. (C) R^2^ and RMSE values for the Dose model (x) and the Bliss model (o). The full names of the drugs are in Table 2. The response curves presented here are the average of two biological replicates.

We find that the combinations show synergy and antagonism—they differ from the Bliss model predictions (Fig 4). For example, the synergistic combination Bedaquiline(1), Clofazimine(2), Ethionamide(3) and Isoniazid(5) provided reduction of 40% in dose needed to achieve a 50% reduction in growth, g = 0.5 relative to the Bliss model (and also relative to the Loewe model that gives almost identical prediction to the Bliss in this case).

The dose model provides excellent prediction for all measured combinations, with R^2^ values exceeding 0.9 and RMSE of about 0.06 (Fig 4).

## Discussion

We find that the dose model of ref [22] provides excellent predictions for combinations of up to ten drugs in *E. coli* and *M. tuberculosis*, based on measurements of drug pairs. Previously this model was tested only up to combinations of four drugs.

Because the dose-model uses information from pairwise interactions, it provides great efficiency in the search for ultra-high-order drug synergies. The efficiency stems from the fact that there is a quadratic number of pairwise combinations n(n-1)/2, which is much less than the exponential number of all possible combinations among n drugs, 2^n^. Therefore, conducting pairwise experiments and making predictions on high-order combinations provides an exponential increase in efficiency. For example, there are around 20 antibiotics specifically effective against *M. tuberculosis*. There are 190 pairwise interactions among these antibiotics, whereas there are 1 million different combinations involving any number of drugs. When one adds the number of possible doses, the efficiency of using pairs increases even more.

The use of the present approach may help to discover ultra-high-order antibiotic synergies against *M*. *tuberculosis*, with lower toxicity and higher efficacy than current treatment. Combinations may also slow down antibiotic resistance, because multiple bottlenecks are introduced by drugs with different targets. In this study, we identified one such four-antibiotic synergy against *M*. *tuberculosis* (Bdq+Clz+Eta+Inh).

The present results bear on the question of high-order interactions beyond pairs. High-order drug interactions can be considered as an amalgam of all possible interactions among the constituents [3]–[5], [14]. For example, the observed interaction of the combination A+B+C is the result of three pairwise interactions (A+B, A+C, B+C) and one emergent interaction among all three drugs (A+B+C). In this setting, the emergent interaction is a quantity that cannot be predicted using pairwise interactions. Thus, the emergent interactions depend on the null model used, such as the Bliss model, Lowe model or Isserlis-like formula used in [3]. For example, using different null models, several previous studies found prevalent emergent interactions[3]–[5], [14]. The present finding that pairwise interactions can successfully predict high-order interactions using the dose-model is in agreement with the hypothesis that emergent interactions are rare.

The majority of drug interactions in high-order combinations studied here were antagonistic compared to Bliss and Loewe. This antagonism follows from the fact that most pairwise interactions are antagonistic in this study, as well as in previous studies [29]. In cases where antagonism is common, prediction methods are especially important to discover rare synergies in the vast space of antagonistic ultra-high-order combinations.

## Materials and methods

Strains and cultures: *E*. *coli* MG1655 strain and a pantothenate and leucine auxotrophic strain of *M*. *tuberculosis* H37Rv were used for experiments[35]. *E*. *coli* cells were grown overnight to saturation in LB, *M*. *tuberculosis* cells were grown to mid-log phase in 7H9 medium supplemented with 0.05% Tween 80, 0.2% glycerol, 10% BBL Middlebrook OADC enrichment (VWR), 50μg/ml leucine, and 24μg/ml pantothenate.

Growth inhibition assays: Drugs were dissolved in DMSO and stored at -20°C. The top concentration used for each drug is given in Table 1. Assays were performed in clear, flat bottom 384-well microplates by dispensing nanoliter volumes of drugs using a digital drug dispenser (D300e Digital Dispenser, HP). Dispense locations were randomized within each plate in order to minimize plate position effect. *E*. *coli* and *M*. *tuberculosis* cells were diluted using fresh media to an OD of 0.01 and 0.05, respectively. 50 μL diluted cells were added to each well. Plates were sealed with aluminum plate seals and incubated without shaking at 37°C. OD600 for *E*. *coli* or *M*. *tuberculosis* plates were measured after 12 hours or 5 days, respectively (see example of growth curves in S9 Fig). Each measurement was done in two or three biological replicates.

Data analysis: Using the randomization map from the digital dispense software, plate measurement data were reconstituted and analyzed (MATLAB, Mathworks) as described above.

Single-drug dose-response curves: The single-drug dose-response curves were fitted by Hill curves ([24], [25]), characterized by a steepness parameter (Hill coefficient) *n*_*i*_ and a halfway point *D*_0*i*_ equal to the drug concentration of 50% effect:

$$g_{i}=\frac{1}{1+{\left(D_{i}/D_{0i}\right)}^{n}}$$

Where *g*_*i*_,*D*_*i*_ are the effect (growth inhibition) and the dose of drug *i*.

Bliss Independence Model ([27], [28]): The model assumes that the effect of drug combination is the product of the single-drug effects:

$$g_{1..n}=g_{1}(D_{1})∙g_{2}(D_{2})\cdots g_{n}(D_{n})$$

Where *g*_*i*_, is the single-drug effect of drug *i* and *D*_*i*_ is the dose of drug *i*.

Dose Model: The dose model [22] is an extension of the Bliss model to include the effect of drug interactions. It is based on the product of the effects of all drugs in the cocktail, but not at their true doses but rather at effective doses that differ from the true doses due to interactions with the other drugs in the cocktail. This interaction is modeled by introducing Michaelis-Menten-like interaction terms between drug pairs, where drug *i* changes the effective dose of drug *j* (and vice versa):

$$g_{ij}=g_{i}(D_{ieff})∙g_{j}(D_{jeff})$$

$$D_{ieff}=D_{i}{\left(1+a_{ij}\frac{D_{jeff}/D_{0j}}{1+D_{jeff}/D_{0j}}\right)}^{-1}D_{jeff}=D_{j}{\left(1+a_{ji}\frac{D_{ieff}/D_{0i}}{1+D_{ieff}/D_{0i}}\right)}^{-1}$$

Where *g*_*i*_,*g*_*j*_ are the single-drug dose responses (described by Hill curves); *g*_*ij*_ is the pair response of drug *i* and drug *j*; *D*_*i*_,*D*_*j*_ are the doses of drug *i* and drug *j* in the cocktail; *D*_*ieff*_,*D*_*jeff*_ are the effective doses; *D*_0*i*_,*D*_0*j*_ are the halfway doses of drug *i* and drug *j*; *a*_*ij*_,*a*_*ji*_ are the interaction parameters.

Using the single and pair dose response data the interaction parameters *a*_*ij*_,*a*_*ji*_ are fitted using MATLAB “fit” function. The effect of any drug combination is predicted by the dose model formula, by assuming the effects of the drugs on each other’s effective concentration are multiplicative:

$$g_{1..n}=g_{1}(D_{1eff})∙g_{2}(D_{neff})\cdots g_{n}(D_{neff})$$

$$D_{ieff}=D_{i}\prod_{j\neq i}\left(1+a_{ij}\frac{D_{jeff}/D_{0j}}{1+D_{jeff}/D_{0j}}\right)$$

Where the second equation is numerically solved using MATLAB “fmincon” function.

To minimize the effect of experimental noise, we used for predictions single-dose responses and combination-dose responses measured on the same plate.

Loewe additivity Model [32]: The model assumes that the effect of drugs in a combination is additive in the dose, meaning that their combined effect is the same across all combinations that have the same total normalized dose:

$$\sum_{i}\frac{D_{i}}{D_{iX}}=1$$

Where *D*_*i*_,*D*_*j*_ are the doses of drug *i* and drug *j* which gives X% effect in the pair combination and *D*_*iX*_ are the doses that gives X% effect when applied alone.

## Supporting information

S1 FigThe experimental error of the interaction index can be estimated from repeated measurements.(A) The interaction index (I = log2(g12-g1*g2+1)) for pairs of replicate 1 versus replicate 2. The errorbars are two standard deviations (*σ*_*I*_). (B) The distribution of the differences between the two replicates. The line is the pdf of normal disterbution with *σ*_*I*1−*I*2_ = 0.066. This gives estimation of *σ*_*I*_ = 0.047 for one replicate, and $\sigma_{\bar{I}}=0.033$ for the mean value of the two replicates. The threshold for interaction was set to be $2\sigma_{\bar{I}}=0.066$ to give confidence level of 95%.(TIF)

S2 Fig The Bliss multiplicative null model is better than the Loewe null model for high-order combinations against *E. coli*. R^2^ (A) and RMSE values (B) for the Bliss (o), Loewe (*) and Dose(x) Models. As in [29] the Bliss model outperform the Loewe model when the number of drugs increase.
(TIF)

S3 Fig The Bliss multiplicative null model is better than the Loewe null model for high-order combinations against TB. R^2^ (A) and RMSE values (B) for the Bliss (o), Loewe (*) and Dose(x) Models. As in [29] the Bliss model outperforms the Loewe model when the number of drugs increase.
(TIF)

S4 Fig The Dose model error is consistent with the experimental error. (A) Comparison between the distribution of residuals of the dose model, to the distribution of residuals of the experimental repeats compared to each other. In the calculation of the residuals of the experimental repeats we corrected for experimental dose variations and other batch effects in the different repeats using the Bliss model. This means that we calculated the residuals of the deviation from Bliss (g-B) in the different biological repeats. This reduced the experimental RMSE from 0.13 to 0.078. Dose model RMSE is 0.077, very similar to the experimental value. This is in contrast to the RMSE of the Bliss model (B) which is 0.113 and Loewe model which is 0.173. Kolmogorov–Smirnov test finds that the Dose model residual distribution is narrower than the experimental distribution(p = 0.97), and the Bliss and Loewe are wider than the experimental distribution (p<10^−10^)
(TIF)

S5 Fig The distribution of errors for the different combinations is consistent with the experimental error. (A) RMSE values of the dose model for each of the 115 combinations (two to three repeats) compared to the expected RMSE distribution evaluated from the repeated measurements (corrected for dose variation, see S4 Fig). We found that there is no significant difference between the two distributions (Kolmogorov–Smirnov test, p = 0.2) in contrast to the RMSE distribution of the Bliss (B) or Loewe models (C) (Kolmogorov–Smirnov test, p < 10^−8^).
(TIF)

S6 Fig The difference (residual) between the model predictions the experiment for the Dose model (red) and the Bliss model (blue). The error-bars are the standard deviations of 2–3 repeats.
(TIF)

S7 Fig Predicted synergy is rare in cocktails above four drugs, with antagonism being much more common. (A) Histogram of the predicted interaction parameter I (at g = 0.5) for all possible 3–8 antibiotic combinations in this study. (B) The same as (A) but with non-diagonal design. Here I is the average value of 10 random doses with g = 0.4–0.6.
(TIF)

S8 Fig Predicted synergy is common in multi-drug cocktails when the pair interaction is mostly synergistic. Histogram of the interaction parameter I(at g = 0.5) for all possible 3–8 drug combinations of simulated data, in which most of the pair interaction were set to be synergistic.
(TIF)

S9 Fig We used growth curves of *E. coli* and Mtb to choose the end-time of the experiments. (A) For *E. coli* experiments, we recorded the growth for 16 hours, with measurements every 5 minutes. (B) For Mtb experiments, we measured growth in four time points: day 3, 4, 5 and 7. 96 growth curves recorded in each of these experiments are shown with thin colored lines, and the median OD600 at each time point is shown with a thick black line. For *E. coli* and Mtb experiments, cultures reached saturation around 12 hours and 5 days, respectively. We chose these time points as the end-time for our drug interaction screen experiments as this end-point closely corresponds to growth rate.
(TIF)

S1 Table List of all 115 three to ten antibiotic combinations tested against *E. coli* in this study. (DOCX)

S2 Table List of RMSE values for the dose model and for the experimental repeats and their differences for all 115 three to ten antibiotic combinations tested against *E. coli* in this study. The list is ordered by the maximal difference between the model and the experiment. See S1 Table for combination legend.
(DOCX)

S1 Data The data is OD at 600nm. Doses increase linearly; the maximal dose is in Table 1 and Table 2. We conducted five different experiments:
(1) allpairsof10Ecoli—the effect of all pair combinations of ten antibiotics on Ecoli growth.
(2) allpairsof10Mtb—the effect of all pair combinations of ten antibiotics on MTB growth.
(3) Ecoli030817—the effect of 20 three antibiotics combinations on Ecoli growth.
(4) Ecoli0609—the effect of up to five antibiotics combinations on Ecoli growth.
(5) Mtb0609results—the effect of up to five antibiotics combinations on MTB growth.
(6) 0822 –(a) 0822platessetup—the setup of up to 10 antibiotics combinations on Ecoli growth. (b)-(d) 0822rep1/2/3/—the results of three replicates of the experiment.
(ZIP)
