# Supplementary figures and images for: Prediction of ultra-high-order antibiotic combinations based on pairwise interactions
Source: PLoS Comput Biol. 2019 Jan 30;15(1):e1006774. doi: 10.1371/journal.pcbi.1006774 (PMC6370231; doi:10.1371/journal.pcbi.1006774)

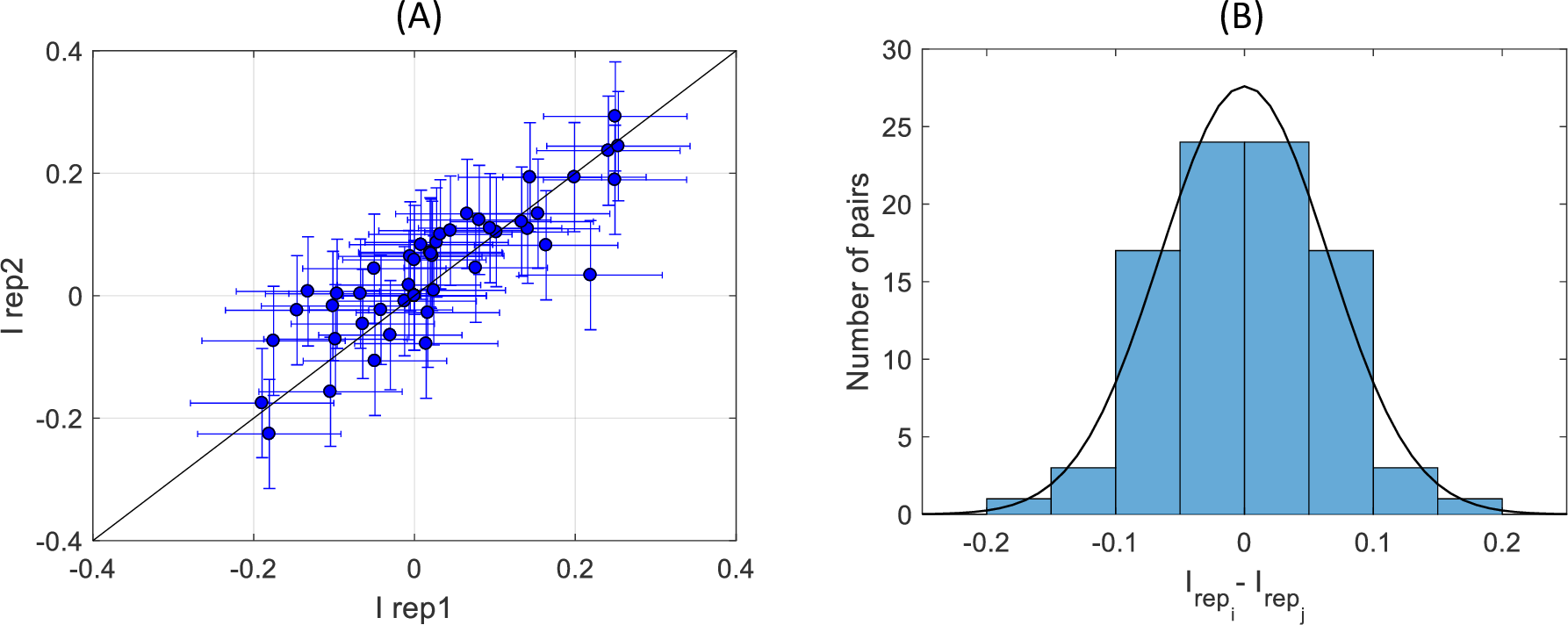

Supplement: S1 Fig — (A) The interaction index (I = log2(g12-g1*g2+1)) for pairs of replicate 1 versus replicate 2. The errorbars are two standard deviations (σI). (B) The distribution of the differences between the two replicates. The line is the pdf of normal disterbution with σI1−I2 = 0.066. This gives estimation of σI = 0.047 for one replicate, and σI¯=0.033 for the mean value of the two replicates. The threshold for interaction was set to be 2σI¯=0.066 to give confidence level of 95%. (TIF) [file pcbi.1006774.s001.tif]

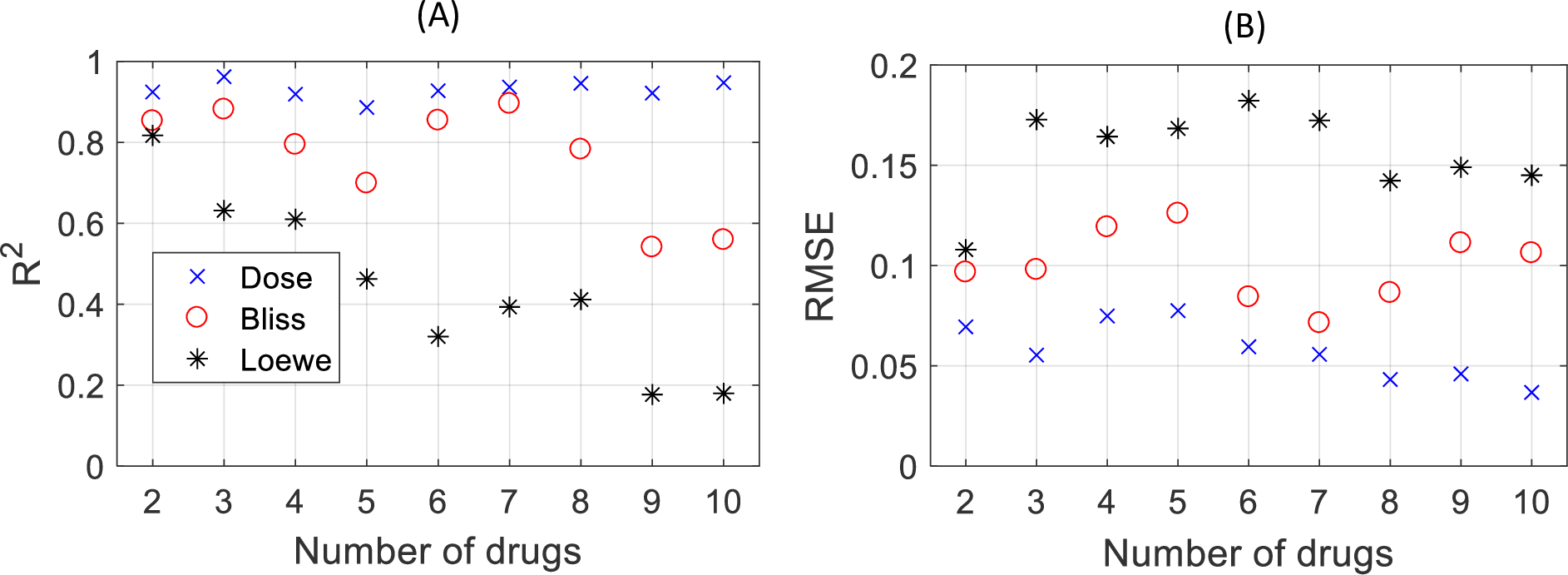

Supplement: S2 Fig — R2 (A) and RMSE values (B) for the Bliss (o), Loewe (*) and Dose(x) Models. As in [29] the Bliss model outperform the Loewe model when the number of drugs increase. (TIF) [file pcbi.1006774.s002.tif]

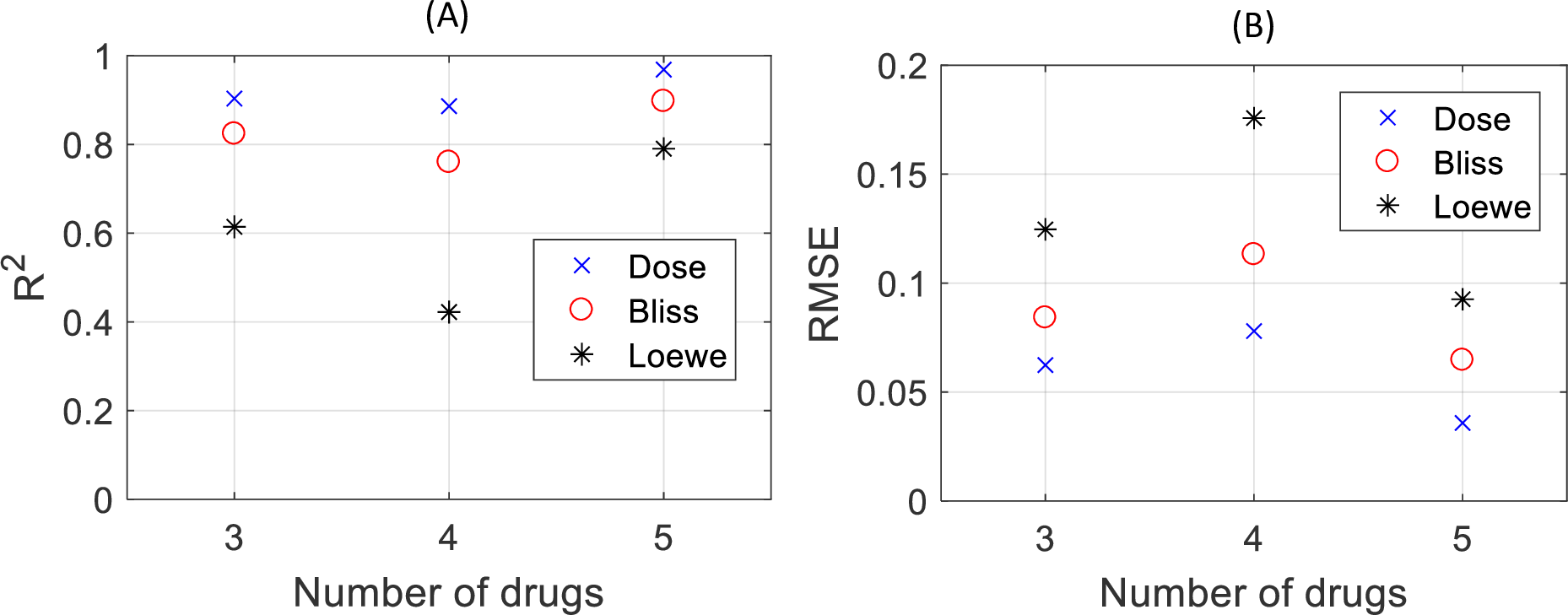

Supplement: S3 Fig — R2 (A) and RMSE values (B) for the Bliss (o), Loewe (*) and Dose(x) Models. As in [29] the Bliss model outperforms the Loewe model when the number of drugs increase. (TIF) [file pcbi.1006774.s003.tif]

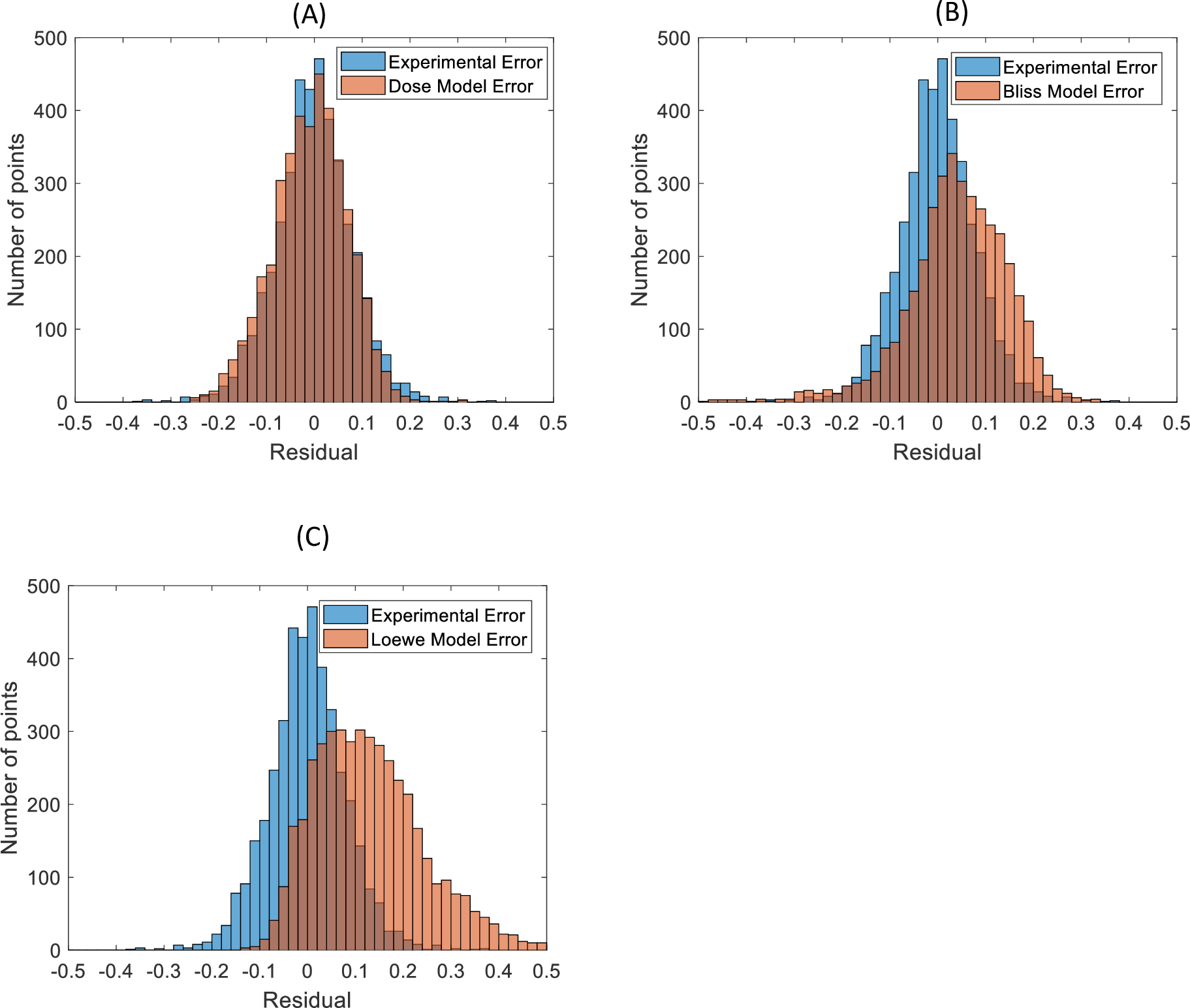

Supplement: S4 Fig — (A) Comparison between the distribution of residuals of the dose model, to the distribution of residuals of the experimental repeats compared to each other. In the calculation of the residuals of the experimental repeats we corrected for experimental dose variations and other batch effects in the different repeats using the Bliss model. This means that we calculated the residuals of the deviation from Bliss (g-B) in the different biological repeats. This reduced the experimental RMSE from 0.13 to 0.078. Dose model RMSE is 0.077, very similar to the experimental value. This is in contrast to the RMSE of the Bliss model (B) which is 0.113 and Loewe model which is 0.173. Kolmogorov–Smirnov test finds that the Dose model residual distribution is narrower than the experimental distribution(p = 0.97), and the Bliss and Loewe are wider than the experimental distribution (p<10−10) (TIF) [file pcbi.1006774.s004.tif]

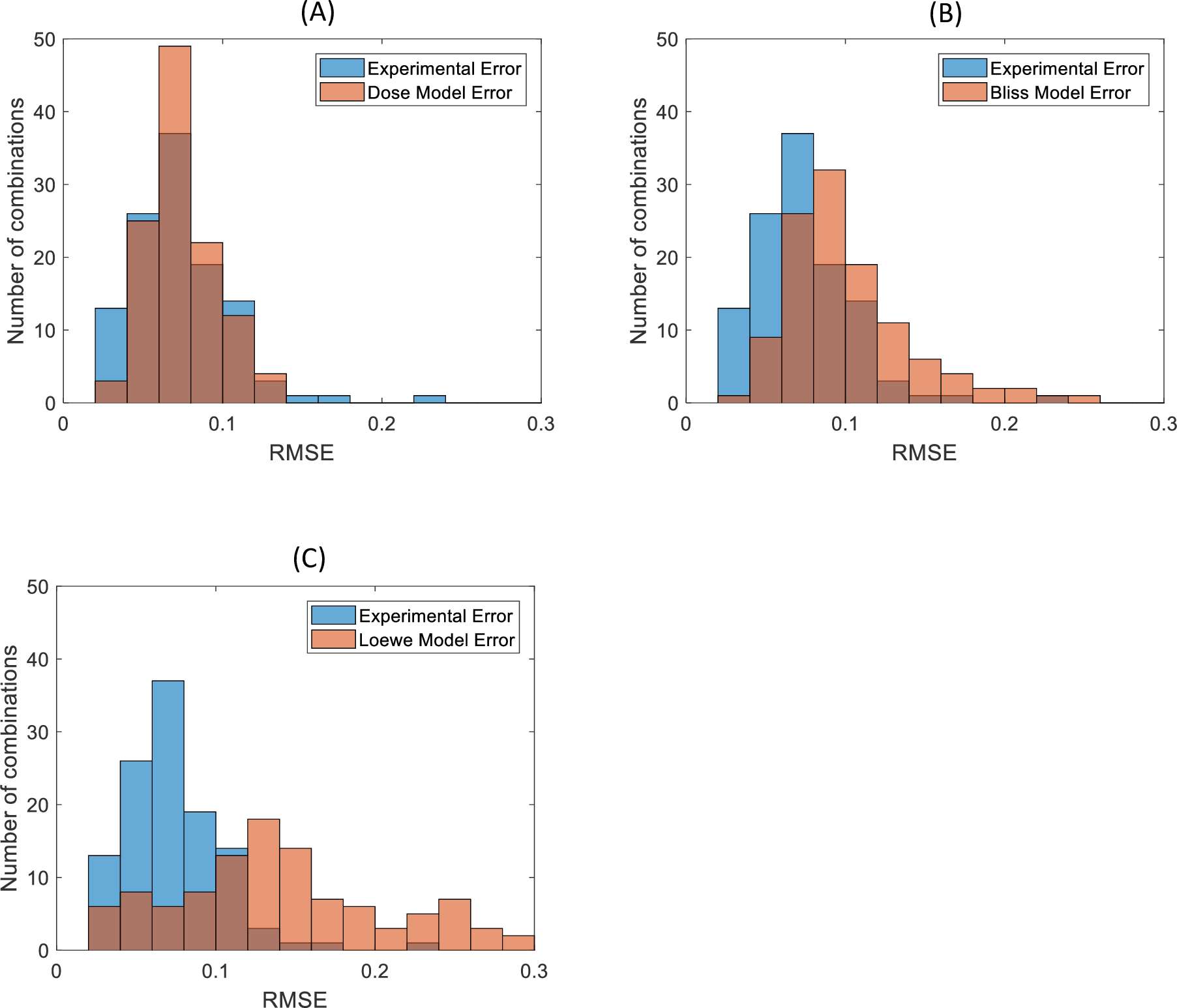

Supplement: S5 Fig — (A) RMSE values of the dose model for each of the 115 combinations (two to three repeats) compared to the expected RMSE distribution evaluated from the repeated measurements (corrected for dose variation, see S4 Fig). We found that there is no significant difference between the two distributions (Kolmogorov–Smirnov test, p = 0.2) in contrast to the RMSE distribution of the Bliss (B) or Loewe models (C) (Kolmogorov–Smirnov test, p < 10−8). (TIF) [file pcbi.1006774.s005.tif]

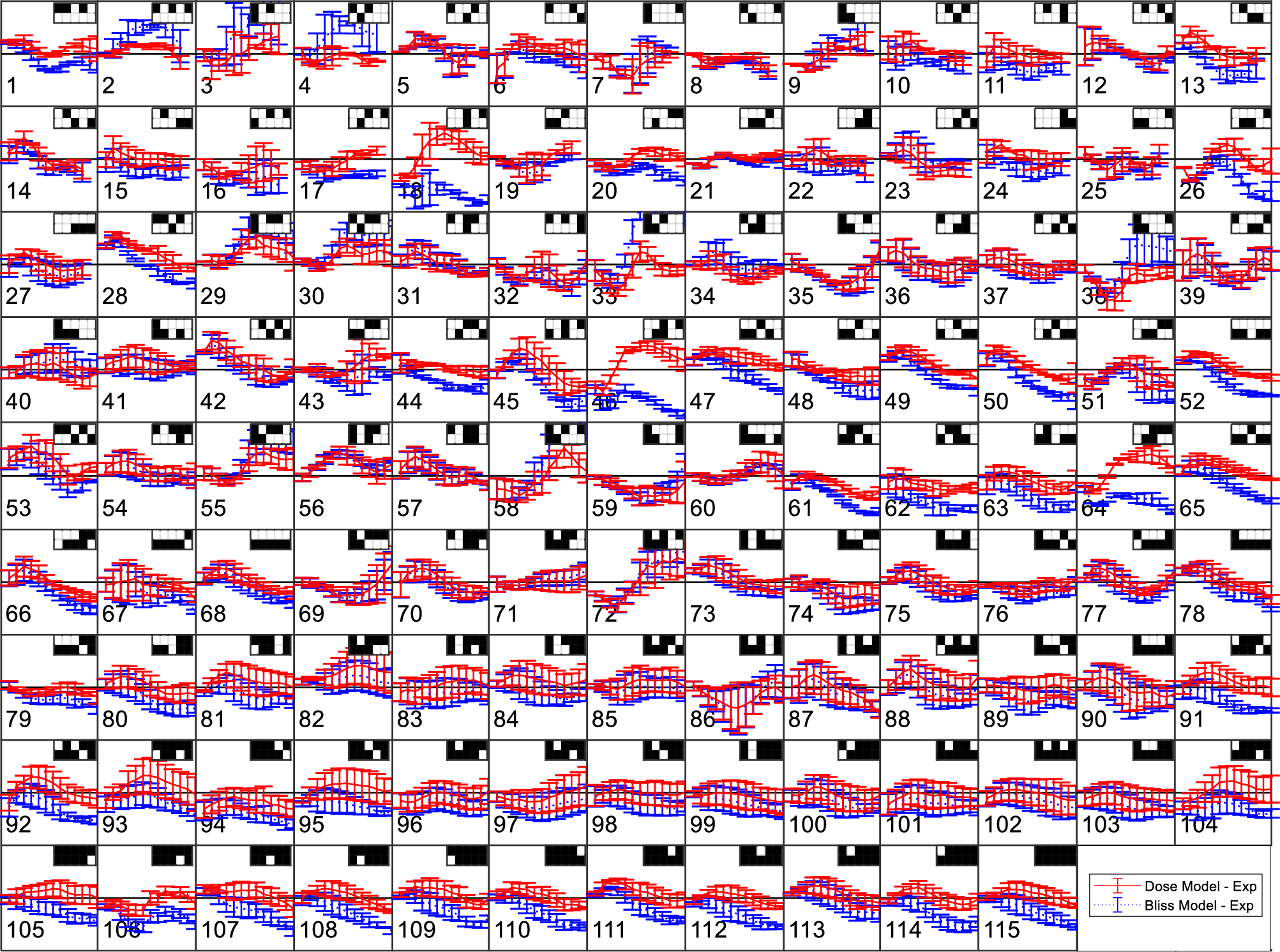

Supplement: S6 Fig — The error-bars are the standard deviations of 2–3 repeats. (TIF) [file pcbi.1006774.s006.tif]

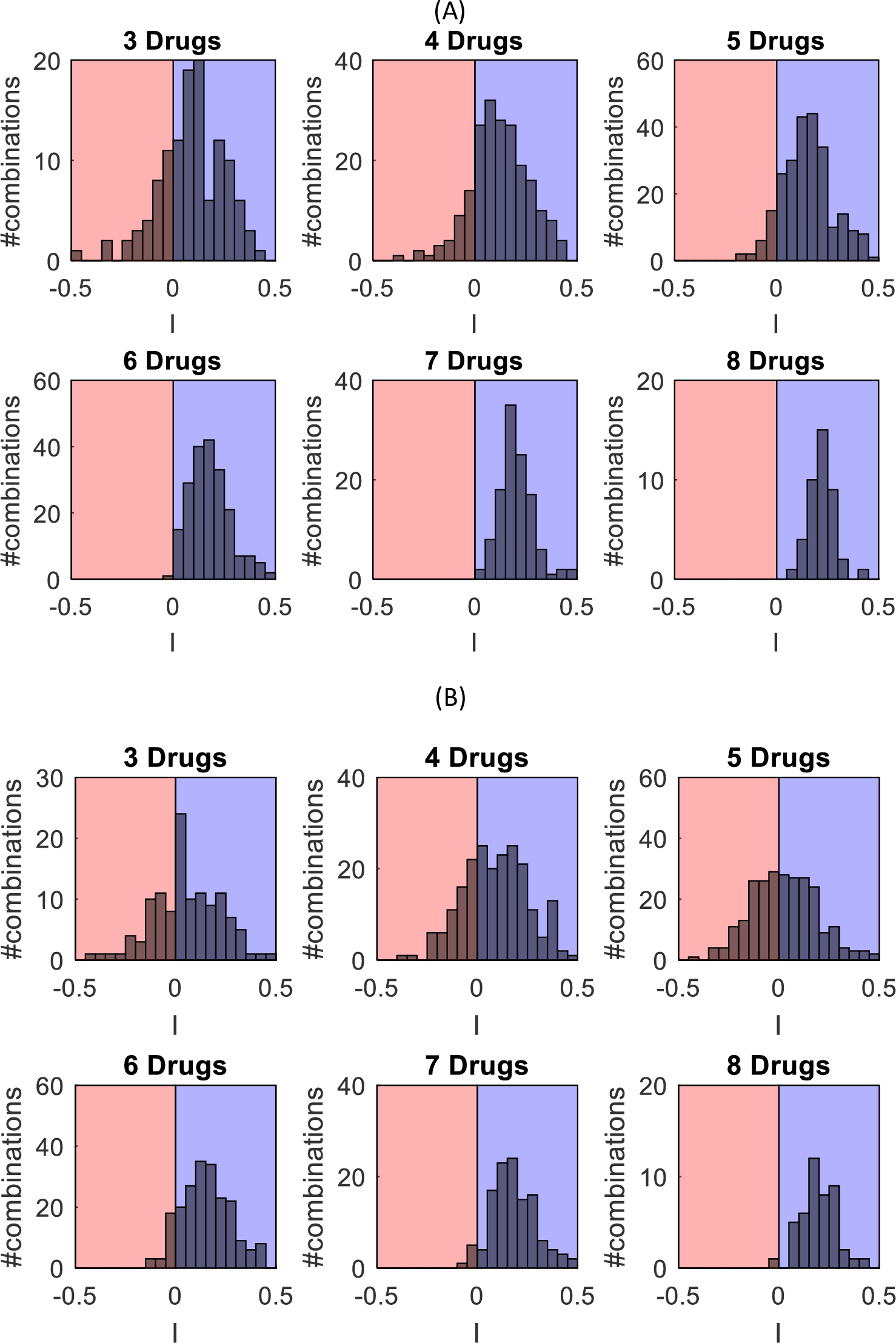

Supplement: S7 Fig — (A) Histogram of the predicted interaction parameter I (at g = 0.5) for all possible 3–8 antibiotic combinations in this study. (B) The same as (A) but with non-diagonal design. Here I is the average value of 10 random doses with g = 0.4–0.6. (TIF) [file pcbi.1006774.s007.tif]

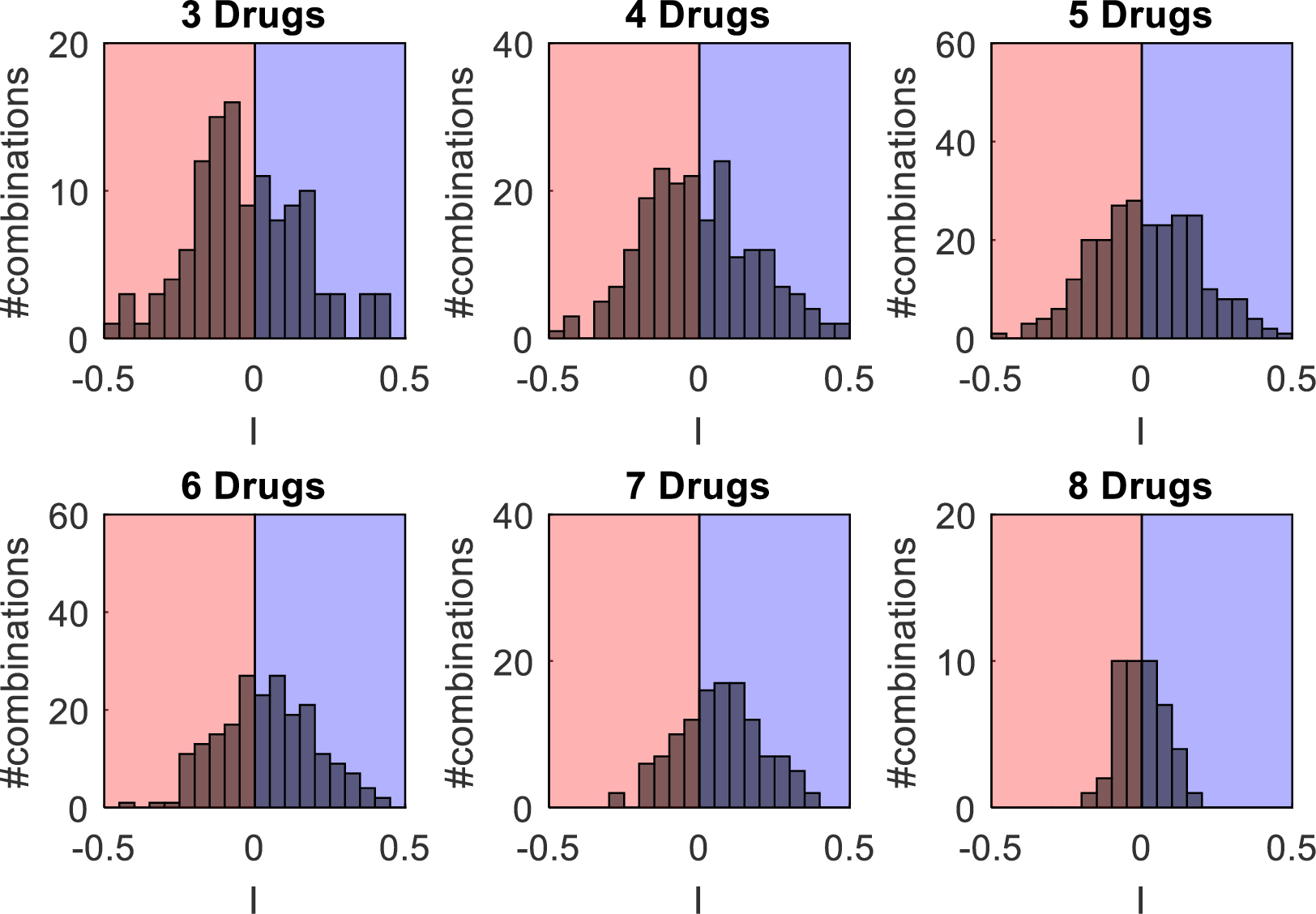

Supplement: S8 Fig — Histogram of the interaction parameter I(at g = 0.5) for all possible 3–8 drug combinations of simulated data, in which most of the pair interaction were set to be synergistic. (TIF) [file pcbi.1006774.s008.tif]

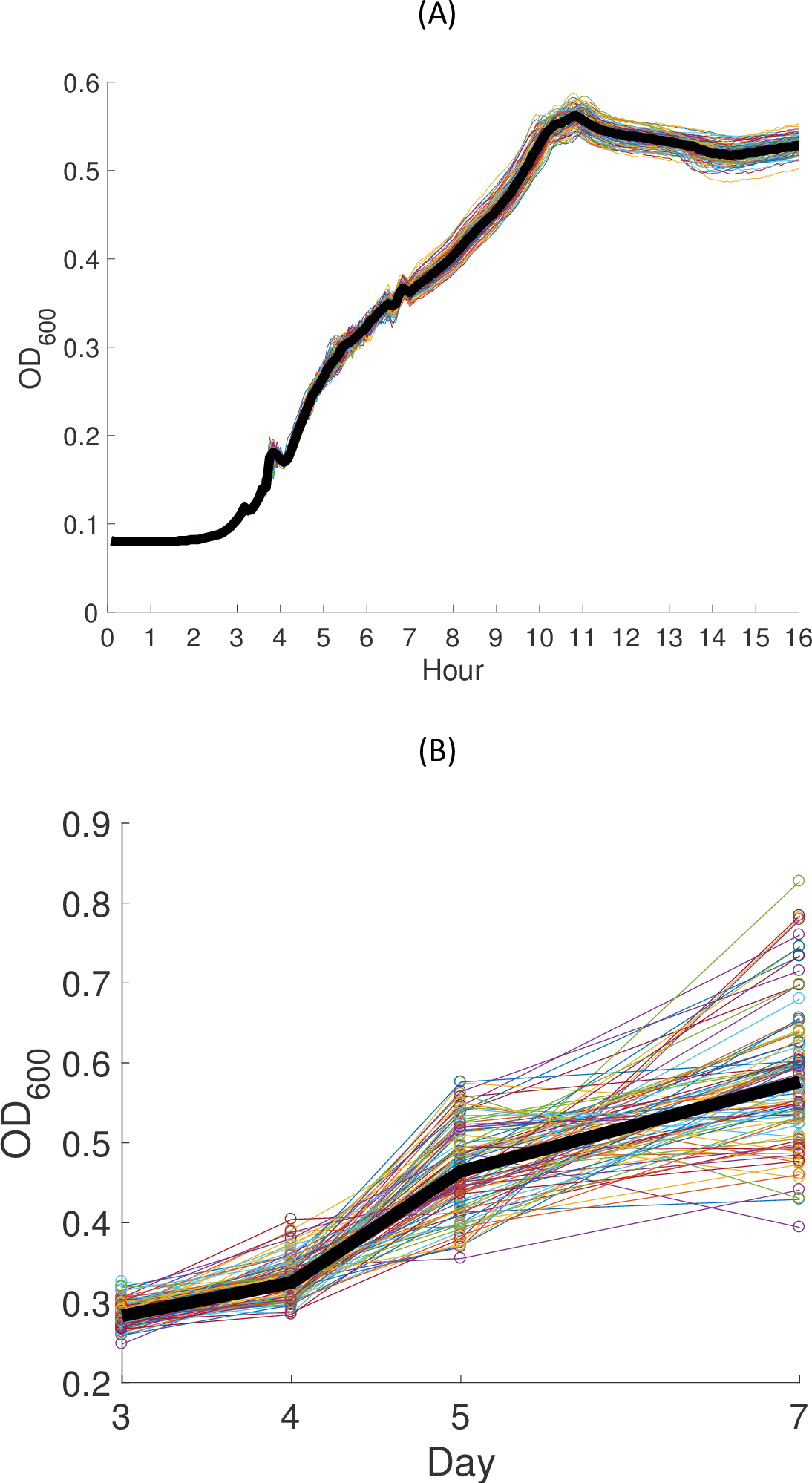

Supplement: S9 Fig — (A) For E. coli experiments, we recorded the growth for 16 hours, with measurements every 5 minutes. (B) For Mtb experiments, we measured growth in four time points: day 3, 4, 5 and 7. 96 growth curves recorded in each of these experiments are shown with thin colored lines, and the median OD600 at each time point is shown with a thick black line. For E. coli and Mtb experiments, cultures reached saturation around 12 hours and 5 days, respectively. We chose these time points as the end-time for our drug interaction screen experiments as this end-point closely corresponds to growth rate. (TIF) [file pcbi.1006774.s009.tif]
