# Supplementary material for: Prediction of ultra-high-order antibiotic combinations based on pairwise interactions
Source: PLoS Comput Biol. 2019 Jan 30;15(1):e1006774. doi: 10.1371/journal.pcbi.1006774 (PMC6370231; doi:10.1371/journal.pcbi.1006774)
Supplement: S1 Table — (DOCX) [file pcbi.1006774.s010.docx]

| 1 | [1,2,4] | 30 | [1,3,4,7] | 59 | [1,5,6,7,10] | 88 | [1,3,5,7,8,9,10] |
| --- | --- | --- | --- | --- | --- | --- | --- |
| 2 | [1,3,5] | 31 | [1,3,4,8] | 60 | [1,6,7,8,10] | 89 | [1,4,5,6,7,8,10] |
| 3 | [1,3,6] | 32 | [1,3,5,10] | 61 | [2,3,6,7,9] | 90 | [1,5,6,7,8,9,10] |
| 4 | [1,3,7] | 33 | [1,3,6,7] | 62 | [2,3,6,8,9] | 91 | [2,3,4,6,7,8,10] |
| 5 | [1,4,8] | 34 | [1,3,7,8] | 63 | [2,6,7,9,10] | 92 | [2,4,5,6,7,8,10] |
| 6 | [1,4,10] | 35 | [1,4,6,7] | 64 | [3,4,5,7,8] | 93 | [1,2,3,4,5,7,8,10] |
| 7 | [1,5,6] | 36 | [1,4,8,9] | 65 | [3,6,7,9,10] | 94 | [1,2,3,5,6,7,8,9] |
| 8 | [1,5,7] | 37 | [1,4,8,10] | 66 | [4,5,7,8,9] | 95 | [1,2,4,5,6,7,8,10] |
| 9 | [1,6,7] | 38 | [1,5,6,7] | 67 | [5,6,7,8,9] | 96 | [1,3,4,5,6,7,8,9] |
| 10 | [2,4,8] | 39 | [1,5,8,9] | 68 | [6,7,8,9,10] | 97 | [1,3,4,5,6,7,8,10] |
| 11 | [2,4,9] | 40 | [1,6,7,8] | 69 | [1,3,4,5,6,7] | 98 | [1,3,4,5,6,7,9,10] |
| 12 | [2,4,10] | 41 | [1,6,7,9] | 70 | [1,3,4,5,8,9] | 99 | [1,3,4,5,6,8,9,10] |
| 13 | [2,8,9] | 42 | [2,4,8,10] | 71 | [1,3,4,6,7,8] | 100 | [1,3,4,5,7,8,9,10] |
| 14 | [2,8,10] | 43 | [3,4,6,7] | 72 | [1,3,5,6,7,8] | 101 | [1,3,4,6,7,8,9,10] |
| 15 | [2,9,10] | 44 | [3,4,7,10] | 73 | [1,3,6,8,9,10] | 102 | [1,3,5,6,7,8,9,10] |
| 16 | [3,5,6] | 45 | [3,5,6,8] | 74 | [1,4,5,6,7,8] | 103 | [1,4,5,6,7,8,9,10] |
| 17 | [3,5,7] | 46 | [3,5,7,8] | 75 | [1,4,6,7,8,9] | 104 | [2,3,4,5,6,7,8,10] |
| 18 | [3,5,8] | 47 | [3,6,7,9] | 76 | [1,4,6,7,8,10] | 105 | [1,2,3,4,5,6,7,8,9] |
| 19 | [3,6,7] | 48 | [3,6,7,10] | 77 | [1,5,7,8,9,10] | 106 | [1,2,3,4,5,6,7,8,10] |
| 20 | [4,5,7] | 49 | [3,6,9,10] | 78 | [1,6,7,8,9,10] | 107 | [1,2,3,4,5,6,7,9,10] |
| 21 | [4,7,8] | 50 | [3,7,9,10] | 79 | [4,5,6,7,8,10] | 108 | [1,2,3,4,5,6,8,9,10] |
| 22 | [4,8,9] | 51 | [4,5,7,8] | 80 | [4,5,6,7,9,10] | 109 | [1,2,3,4,5,7,8,9,10] |
| 23 | [4,8,10] | 52 | [6,7,9,10] | 81 | [1,2,3,5,7,8,10] | 110 | [1,2,3,4,6,7,8,9,10] |
| 24 | [4,9,10] | 53 | [1,2,4,8,10] | 82 | [1,3,4,5,6,7,8] | 111 | [1,2,3,5,6,7,8,9,10] |
| 25 | [5,6,7] | 54 | [1,3,4,5,9] | 83 | [1,3,4,5,6,8,10] | 112 | [1,2,4,5,6,7,8,9,10] |
| 26 | [5,7,8] | 55 | [1,3,4,6,7] | 84 | [1,3,4,5,6,9,10] | 113 | [1,3,4,5,6,7,8,9,10] |
| 27 | [8,9,10] | 56 | [1,3,4,6,8] | 85 | [1,3,4,6,7,8,10] | 114 | [2,3,4,5,6,7,8,9,10] |
| 28 | [1,2,4,8] | 57 | [1,3,4,8,9] | 86 | [1,3,5,6,7,8,10] | 115 | [1,2,3,4,5,6,7,8,9,10] |
| 29 | [1,3,4,6] | 58 | [1,3,5,6,7] | 87 | [1,3,5,6,8,9,10] |  |  |

| # | Model | Exp | Model  -  Exp | # | Model | Exp | Model  -  Exp | # | Model | Exp | Model  –  Exp |
| --- | --- | --- | --- | --- | --- | --- | --- | --- | --- | --- | --- |
| 46 | 0.12 | 0.03 | 0.08 | 78 | 0.07 | 0.06 | 0.02 | 88 | 0.09 | 0.11 | -0.02 |
| 64 | 0.10 | 0.03 | 0.07 | 18 | 0.11 | 0.09 | 0.02 | 11 | 0.06 | 0.08 | -0.02 |
| 28 | 0.11 | 0.04 | 0.07 | 62 | 0.08 | 0.06 | 0.01 | 96 | 0.07 | 0.09 | -0.02 |
| 56 | 0.10 | 0.03 | 0.07 | 69 | 0.09 | 0.08 | 0.01 | 103 | 0.07 | 0.09 | -0.02 |
| 45 | 0.10 | 0.04 | 0.06 | 14 | 0.08 | 0.07 | 0.01 | 99 | 0.07 | 0.09 | -0.02 |
| 59 | 0.11 | 0.05 | 0.06 | 73 | 0.07 | 0.06 | 0.01 | 100 | 0.07 | 0.09 | -0.03 |
| 61 | 0.08 | 0.02 | 0.06 | 106 | 0.06 | 0.04 | 0.01 | 40 | 0.07 | 0.09 | -0.03 |
| 49 | 0.08 | 0.03 | 0.05 | 23 | 0.06 | 0.05 | 0.01 | 98 | 0.06 | 0.08 | -0.03 |
| 53 | 0.13 | 0.08 | 0.05 | 91 | 0.07 | 0.06 | 0.01 | 39 | 0.09 | 0.12 | -0.03 |
| 47 | 0.09 | 0.04 | 0.05 | 68 | 0.07 | 0.06 | 0.01 | 97 | 0.08 | 0.11 | -0.03 |
| 32 | 0.09 | 0.04 | 0.05 | 20 | 0.04 | 0.04 | 0.01 | 36 | 0.07 | 0.10 | -0.03 |
| 72 | 0.12 | 0.08 | 0.04 | 17 | 0.05 | 0.04 | 0.01 | 85 | 0.07 | 0.10 | -0.03 |
| 42 | 0.11 | 0.06 | 0.04 | 1 | 0.06 | 0.05 | 0.01 | 89 | 0.06 | 0.09 | -0.03 |
| 52 | 0.07 | 0.03 | 0.04 | 104 | 0.08 | 0.08 | 0.01 | 37 | 0.05 | 0.08 | -0.03 |
| 29 | 0.11 | 0.07 | 0.04 | 92 | 0.08 | 0.07 | 0.01 | 67 | 0.07 | 0.11 | -0.03 |
| 60 | 0.06 | 0.02 | 0.04 | 38 | 0.13 | 0.12 | 0.01 | 101 | 0.07 | 0.10 | -0.03 |
| 50 | 0.08 | 0.05 | 0.03 | 57 | 0.08 | 0.07 | 0.01 | 86 | 0.11 | 0.14 | -0.03 |
| 65 | 0.07 | 0.04 | 0.03 | 95 | 0.08 | 0.08 | 0.01 | 83 | 0.08 | 0.11 | -0.03 |
| 111 | 0.07 | 0.04 | 0.03 | 26 | 0.06 | 0.06 | 0.00 | 102 | 0.07 | 0.11 | -0.03 |
| 55 | 0.10 | 0.08 | 0.02 | 75 | 0.06 | 0.05 | 0.00 | 33 | 0.08 | 0.11 | -0.04 |
| 35 | 0.09 | 0.07 | 0.02 | 31 | 0.06 | 0.06 | 0.00 | 16 | 0.08 | 0.12 | -0.04 |
| 93 | 0.10 | 0.08 | 0.02 | 70 | 0.07 | 0.07 | 0.00 | 7 | 0.08 | 0.12 | -0.04 |
| 8 | 0.06 | 0.04 | 0.02 | 105 | 0.06 | 0.05 | 0.00 | 90 | 0.09 | 0.13 | -0.05 |
| 66 | 0.08 | 0.06 | 0.02 | 110 | 0.05 | 0.05 | 0.00 | 9 | 0.05 | 0.11 | -0.05 |
| 94 | 0.09 | 0.07 | 0.02 | 21 | 0.03 | 0.03 | 0.00 | 2 | 0.05 | 0.10 | -0.06 |
| 74 | 0.08 | 0.06 | 0.02 | 30 | 0.08 | 0.08 | 0.00 | 22 | 0.07 | 0.13 | -0.06 |
| 13 | 0.08 | 0.06 | 0.02 | 108 | 0.06 | 0.06 | 0.00 | 4 | 0.05 | 0.17 | -0.12 |
| 82 | 0.13 | 0.11 | 0.02 | 107 | 0.06 | 0.06 | 0.00 | 3 | 0.10 | 0.22 | -0.13 |
| 58 | 0.10 | 0.08 | 0.02 | 109 | 0.06 | 0.06 | 0.00 |  |  |  |  |
