# Supplementary material for: Prediction of ultra-high-order antibiotic combinations based on pairwise interactions
Source: PLoS Comput Biol. 2019 Jan 30;15(1):e1006774. doi: 10.1371/journal.pcbi.1006774 (PMC6370231; doi:10.1371/journal.pcbi.1006774)
Supplement: S2 Table — The list is ordered by the maximal difference between the model and the experiment. See S1 Table for combination legend. (DOCX) [file pcbi.1006774.s011.docx]

| # | Model | Exp | Model  -  Exp | # | Model | Exp | Model  -  Exp | # | Model | Exp | Model  –  Exp |
| --- | --- | --- | --- | --- | --- | --- | --- | --- | --- | --- | --- |
| 46 | 0.12 | 0.03 | 0.08 | 78 | 0.07 | 0.06 | 0.02 | 88 | 0.09 | 0.11 | -0.02 |
| 64 | 0.10 | 0.03 | 0.07 | 18 | 0.11 | 0.09 | 0.02 | 11 | 0.06 | 0.08 | -0.02 |
| 28 | 0.11 | 0.04 | 0.07 | 62 | 0.08 | 0.06 | 0.01 | 96 | 0.07 | 0.09 | -0.02 |
| 56 | 0.10 | 0.03 | 0.07 | 69 | 0.09 | 0.08 | 0.01 | 103 | 0.07 | 0.09 | -0.02 |
| 45 | 0.10 | 0.04 | 0.06 | 14 | 0.08 | 0.07 | 0.01 | 99 | 0.07 | 0.09 | -0.02 |
| 59 | 0.11 | 0.05 | 0.06 | 73 | 0.07 | 0.06 | 0.01 | 100 | 0.07 | 0.09 | -0.03 |
| 61 | 0.08 | 0.02 | 0.06 | 106 | 0.06 | 0.04 | 0.01 | 40 | 0.07 | 0.09 | -0.03 |
| 49 | 0.08 | 0.03 | 0.05 | 23 | 0.06 | 0.05 | 0.01 | 98 | 0.06 | 0.08 | -0.03 |
| 53 | 0.13 | 0.08 | 0.05 | 91 | 0.07 | 0.06 | 0.01 | 39 | 0.09 | 0.12 | -0.03 |
| 47 | 0.09 | 0.04 | 0.05 | 68 | 0.07 | 0.06 | 0.01 | 97 | 0.08 | 0.11 | -0.03 |
| 32 | 0.09 | 0.04 | 0.05 | 20 | 0.04 | 0.04 | 0.01 | 36 | 0.07 | 0.10 | -0.03 |
| 72 | 0.12 | 0.08 | 0.04 | 17 | 0.05 | 0.04 | 0.01 | 85 | 0.07 | 0.10 | -0.03 |
| 42 | 0.11 | 0.06 | 0.04 | 1 | 0.06 | 0.05 | 0.01 | 89 | 0.06 | 0.09 | -0.03 |
| 52 | 0.07 | 0.03 | 0.04 | 104 | 0.08 | 0.08 | 0.01 | 37 | 0.05 | 0.08 | -0.03 |
| 29 | 0.11 | 0.07 | 0.04 | 92 | 0.08 | 0.07 | 0.01 | 67 | 0.07 | 0.11 | -0.03 |
| 60 | 0.06 | 0.02 | 0.04 | 38 | 0.13 | 0.12 | 0.01 | 101 | 0.07 | 0.10 | -0.03 |
| 50 | 0.08 | 0.05 | 0.03 | 57 | 0.08 | 0.07 | 0.01 | 86 | 0.11 | 0.14 | -0.03 |
| 65 | 0.07 | 0.04 | 0.03 | 95 | 0.08 | 0.08 | 0.01 | 83 | 0.08 | 0.11 | -0.03 |
| 111 | 0.07 | 0.04 | 0.03 | 26 | 0.06 | 0.06 | 0.00 | 102 | 0.07 | 0.11 | -0.03 |
| 55 | 0.10 | 0.08 | 0.02 | 75 | 0.06 | 0.05 | 0.00 | 33 | 0.08 | 0.11 | -0.04 |
| 35 | 0.09 | 0.07 | 0.02 | 31 | 0.06 | 0.06 | 0.00 | 16 | 0.08 | 0.12 | -0.04 |
| 93 | 0.10 | 0.08 | 0.02 | 70 | 0.07 | 0.07 | 0.00 | 7 | 0.08 | 0.12 | -0.04 |
| 8 | 0.06 | 0.04 | 0.02 | 105 | 0.06 | 0.05 | 0.00 | 90 | 0.09 | 0.13 | -0.05 |
| 66 | 0.08 | 0.06 | 0.02 | 110 | 0.05 | 0.05 | 0.00 | 9 | 0.05 | 0.11 | -0.05 |
| 94 | 0.09 | 0.07 | 0.02 | 21 | 0.03 | 0.03 | 0.00 | 2 | 0.05 | 0.10 | -0.06 |
| 74 | 0.08 | 0.06 | 0.02 | 30 | 0.08 | 0.08 | 0.00 | 22 | 0.07 | 0.13 | -0.06 |
| 13 | 0.08 | 0.06 | 0.02 | 108 | 0.06 | 0.06 | 0.00 | 4 | 0.05 | 0.17 | -0.12 |
| 82 | 0.13 | 0.11 | 0.02 | 107 | 0.06 | 0.06 | 0.00 | 3 | 0.10 | 0.22 | -0.13 |
| 58 | 0.10 | 0.08 | 0.02 | 109 | 0.06 | 0.06 | 0.00 |  |  |  |  |
